# Plant Extracellular Vesicles and Nanovesicles: Focus on Secondary Metabolites, Proteins and Lipids with Perspectives on Their Potential and Sources

**DOI:** 10.3390/ijms22073719

**Published:** 2021-04-02

**Authors:** Eric Woith, Gea Guerriero, Jean-Francois Hausman, Jenny Renaut, Céline C. Leclercq, Christoph Weise, Sylvain Legay, Alexander Weng, Matthias F. Melzig

**Affiliations:** 1 Freie Universität Berlin, Institute of Pharmacy, Pharmaceutical Biology, Dahlem Center of Plant Sciences, Königin-Luise-Str. 2+4, D-14195 Berlin, Germany; e.woith@fu-berlin.de (E.W.); matthias.melzig@fu-berlin.de (M.F.M.); 2 Luxembourg Institute of Science and Technology, Environmental Research and Innovation Department, 5 rue Bommel, L-4940 Hautcharage, Luxembourg; jean-francois.hausman@list.lu (J.-F.H.); sylvain.legay@list.lu (S.L.); 3 Luxembourg Institute of Science and Technology, Environmental Research and Innovation Department, 41 Rue du Brill, L-4422 Belvaux, Luxembourg; jenny.renaut@list.lu (J.R.); celine.leclercq@list.lu (C.C.L.); 4 Freie Universität Berlin, Institute of Chemistry and Biochemistry, Core Facility BioSupraMol, Thielallee 63, D-14195 Berlin, Germany; chris.weise@biochemie.fu-berlin.de

**Keywords:** extracellular vesicles, plant nanovesicles, exosome-like nanoparticles, in vitro plant cell culture, lipids, metabolomics, proteomics

## Abstract

While human extracellular vesicles (EVs) have attracted a big deal of interest and have been extensively characterized over the last years, plant-derived EVs and nanovesicles have earned less attention and have remained poorly investigated. Although a series of investigations already revealed promising beneficial health effects and drug delivery properties, adequate (pre)clinical studies are rare. This fact might be caused by a lack of sources with appropriate qualities. Our study introduces plant cell suspension culture as a new and well controllable source for plant EVs. Plant cells, cultured in vitro, release EVs into the growth medium which could be harvested for pharmaceutical applications. In this investigation we characterized EVs and nanovesicles from distinct sources. Our findings regarding secondary metabolites indicate that these might not be packaged into EVs in an active manner but enriched in the membrane when lipophilic enough, since apparently lipophilic compounds were associated with nanovesicles while more hydrophilic structures were not consistently found. In addition, protein identification revealed a possible explanation for the mechanism of EV cell wall passage in plants, since cell wall hydrolases like 1,3-*β*-glucosidases, pectinesterases, polygalacturonases, *β*-galactosidases and *β*-xylosidase/*α*-L-arabinofuranosidase 2-like are present in plant EVs and nanovesicles which might facilitate cell wall transition. Further on, the identified proteins indicate that plant cells secrete EVs using similar mechanisms as animal cells to release exosomes and microvesicles.

## 1. Introduction

Plant nanovesicles are closely related to mammalian extracellular vesicles (EVs) but, compared to the latter, they have been relegated to the background. However, scientific interest has been increasingly paid to the plant equivalent of exosomes and microvesicles lately [1,2].

**Figure 1:**
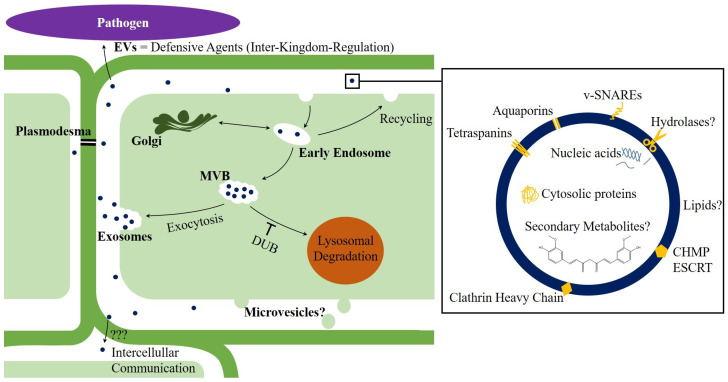
Scheme of current issues in plant nanovesicle research. The illustrated mechanisms of extracellular vesicle (EV) formation can probably be transferred from mammals [6,7] to eukaryotic cells in general. While evidence for the release of exosomes has been reported, it remains unknown whether plant cells also secrete plasma membrane derived microvesicles. Apparently, EVs from plants exert inhibitory effects against pathogenic microorganisms [8,9,10,11] and spread information to other cells, although it has not yet been conclusively elucidated how EVs pass through cell walls. Like other EVs, plant nanovesicles carry distinct nucleic acids. Proteomic analysis of plant nanovesicles revealed the repeated identification of cytosolic proteins (e.g., heat shock proteins), as well as membrane-associated proteins. It remains puzzling, whether cell wall hydrolases are membrane associated and if they enable cell wall passage. Also to be clarified are the questions why and to which extent lipid compositions can vary, as well as if secondary metabolites are packaged into nanovesicles. CHMP: charged multivesicular body protein; DUB: deubiquitinases; ESCRT: endosomal-sorting complex required for transport; EV: extracellular vesicle; MVB: multivesicular body; v-SNARE: vesicular soluble *N*-ethylmaleimide-sensitive-factor attachment receptor.

Structure and density of plant nanovesicles are comparable with exosomes from mammals [3]. Also, multivesicular bodies (MVBs) [4] and apoptotic bodies [5] have been observed in plants and even though we found no reports of plasma membrane-derived microvesicles, we assume that the mechanisms of EV formation [6] can be transferred from the animal kingdom to plants, or even to eukaryotes in general. However, not all of those “exosome-like nanoparticles”, that can be isolated from (homogenized) plant materials like juices, are certainly of extracellular origin. We therefore recommend using the term “nanovesicles” for nanometer-scaled (1–999 nm) membrane-delimited vesicles, as long as the selected raw material does not ensure that EVs are concentrated. For instance, isolated nanovesicles from apoplastic fluid can be assumed to be EVs. The scheme in Figure 1 provides an overview on present key issues in plant nanovesicle research.

Current research regarding plant nanovesicles deals with general properties [3,12,13,14], host-versus-pathogen-interactions [8,11,15,16,17] and health-beneficial effects [18,19,20,21,22,23,24]. Another approach to use the advantageous properties of plant nanovesicles can be to foster the use of these small non-coding RNA (sRNA)-containing vehicles as bio-compatible and sustainable plant protection agents [25]. In order to determine the effects on human health, current preclinical and clinical trials are aiming to prove whether nanovesicles from ginger and aloe improve the condition of polycystic ovary syndrome (NCT03493984), whether grape nanovesicles prevent chemoradiation treatment-associated oral mucositis (NCT01668849), and if plant nanovesicles can effectively deliver curcumin to normal colon tissue and colon tumors (NCT01294072).

Although we have already learned a lot about properties and effects of vesicle subtypes isolated from distinct plant materials, we still neither know for sure how EVs pass through the apoplastic space or cell walls, nor what their function truly is. Considering the inhibitory effects of plant nanovesicles against pathogenic microorganisms and increased vesicle amounts after fungal infections in plants [8,10,11,17,25] together with the diversity of plant nanovesicle lipids [17,18,24,26], one can assume that plant cells produce EVs in order to control the growth of pathogenic microorganisms in case those try to invade the plant and also to address distant host cells, e.g., to improve their immune response. Taking into account that some pests mainly infect particular plants or plant families, it is plausible to imagine that not only the vesicle’s cargo is tuned by the host but also the envelope, to fight such pests as effectively as possible.

Lipid profiles have been shown to vary between different species and we therefore assume, so far, that lipid compositions are species-specific [17], although the extent of variations within the membrane composition in one and the same species remains unknown. Recent results by Berger et al., 2020 indicate that there might also be a family specificity [24]. Anyways, qualitative and quantitative differences of vesicle lipids can hypothetically be utilized to target particular cell types or organs. This assumption is based on the finding that nanovesicles from certain plant species are preferentially taken up by particular cell types [27]. As such, grape exosome-like nanoparticles can target intestinal stem cells [26] while ginger-derived nanovesicles were taken up by hepatocytes and inhibited alcohol-induced liver damage [22]. Likewise, semi-synthetic nanoparticles that have been engineered from grapefruit nanovesicle lipids, were delivered to the brain after nasal application [28] or to liver macrophages after intravenous administration [29]. If research could link the described lipid profiles with specific uptake capabilities into certain cells, we would have a promising tool for targeted drug delivery at hand. In order to get one step closer to that aim, we herein present TLC lipid profiles of some selected plants.

Another aspect of plants’ defense against pathogenic microorganisms might be the packaging of secondary metabolites with anti-microbial activity into EVs. The association of secondary metabolites with plant nanovesicles is also of some relevance regarding the bioavailability of these molecules, as it might increase significantly when secondary metabolites are vesicle-encapsulated. Unfortunately, the few available data on this issue are ambiguous—on the one hand naringin, naringenin [30] and shogaol [22] were found in nanovesicles from grapefruit and ginger. On the other hand are the results of Berger et al., 2020, showing the absence of vitamin C and naringenin in orange nanovesicles [24]. To study the scope of nanovesicle-associated secondary metabolites, we investigated the presence of distinct characteristic alkaline and acidic constituents in nanovesicle-isolates from several pharmaceutically and toxicologically relevant herbal drugs.

Apparently, plant nanovesicles do not exhibit any toxic or immunogenic effects [31], but for the pharmaceutical use of the described nanostructures, a richer knowledge on their properties would help to ensure quality. Identifying characteristic proteins and enzymes that are typically associated with plant nanovesicles could provide both marker proteins for purposes of isolation and analytics, as well as better understanding of (sub-)cellular mechanisms regarding EV formation and distribution.

For the pharmaceutical application of plant nanovesicles, either by themselves or for drug delivery, we need adequate sources for the production of medicinal products with sufficient and constant quality and safety. The establishment of plant cell cultures and their bioprocess optimization could provide a well controllable foundation for this purpose.

In the present article, we show that EVs can be successfully isolated from plant cell culture media following a straightforward procedure that can be easily scaled up to higher volumes. We further provide information on the proteome, as well as on lipids and secondary metabolites of EVs from plant cell culture, as well as of nanovesicles from dried herbs.

## 2. Results

### 2.1. Electron Microscopy

**Figure 2:**
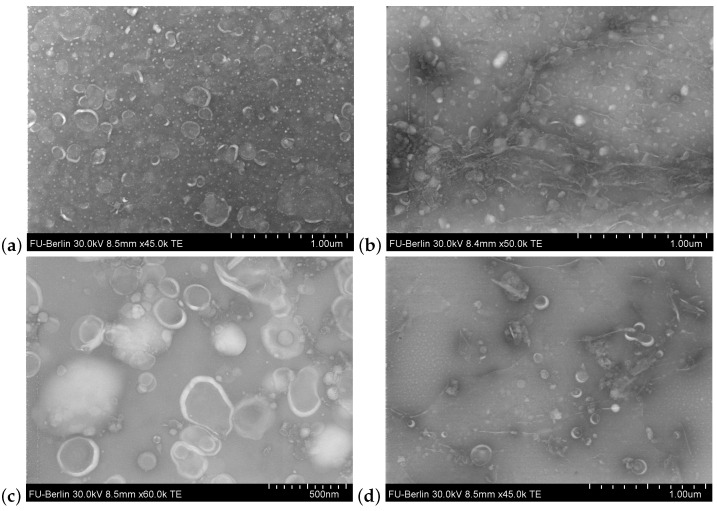
Transmission electron microscopy images of (**a**) extracellular vesicles (EVs) from *Craterostigma plantagineum* Hochst. cell culture medium. (**b**) Nanovesicles from *C. plantagineum* cells. (**c**) Nanovesicles from dried tubers of *Aconitum napellus* L. (**d**) EVs from *Nicotiana tabacum* L. cell culture medium.

Lacking knowledge on marker proteins of plant EVs or nanovesicles at the current state, we imaged the samples that were supposed to contain nanometer-scaled vesicles, using transmission electron microscopy (TEM) in order to confirm the success of the isolation. We observed vesicles with cup-shaped morphology, which has been reported repeatedly for EVs as a resulting artifact of TEM preparation [32]. Morphological differences between EVs and nanovesicles have not been observed, as shown in representative TEM images in Figure 2.

Besides the mainly used isolation technique of differential centrifugation, plant EVs have also been concentrated by tangential flow filtration (TFF, HansaBioMed Life Sciences, Tallinn, Estonia) from cell culture medium (Figure 2d). Since we further noticed that ultrafiltration was not suitable for nanovesicle isolation from herbal drugs due to co-concentration of accompanying polymeric substances, TFF was not utilized for this kind of raw material.

### 2.2. Secondary Metabolites

A crucial factor in secondary metabolite analysis is pH. Consequently, we chose two buffers for our investigations of either acidic or alkaline analytes. When searching for acidic components like curcuminoids, flavonoids or caffeic acid derivatives, a Tris-buffered saline (TBS) pH 8.0 was used (100 mM Tris, 100 mM NaCl, 10 mM EDTA, HCl q.s.) intending to deprotonate acid groups. Due to the resulting charge and thus lipophobicity, these structures should not be able to pass the membrane barrier and would stay either inside or outside the vesicle, depending on their original location.

Conversely, if vesicle samples were investigated for alkaloids, a buffer with acidic pH was used for isolations. We therefore modified the vesicle isolation buffer (VIB) by Rutter and Innes 2017 [13] and used it as follows: 20 mM 2-(*N*-morpholino)ethanesulfonic acid (MES), 100 mM NaCl, 10 mM EDTA, NaOH q.s. (to adjust pH to 5.5).

**Figure 3:**
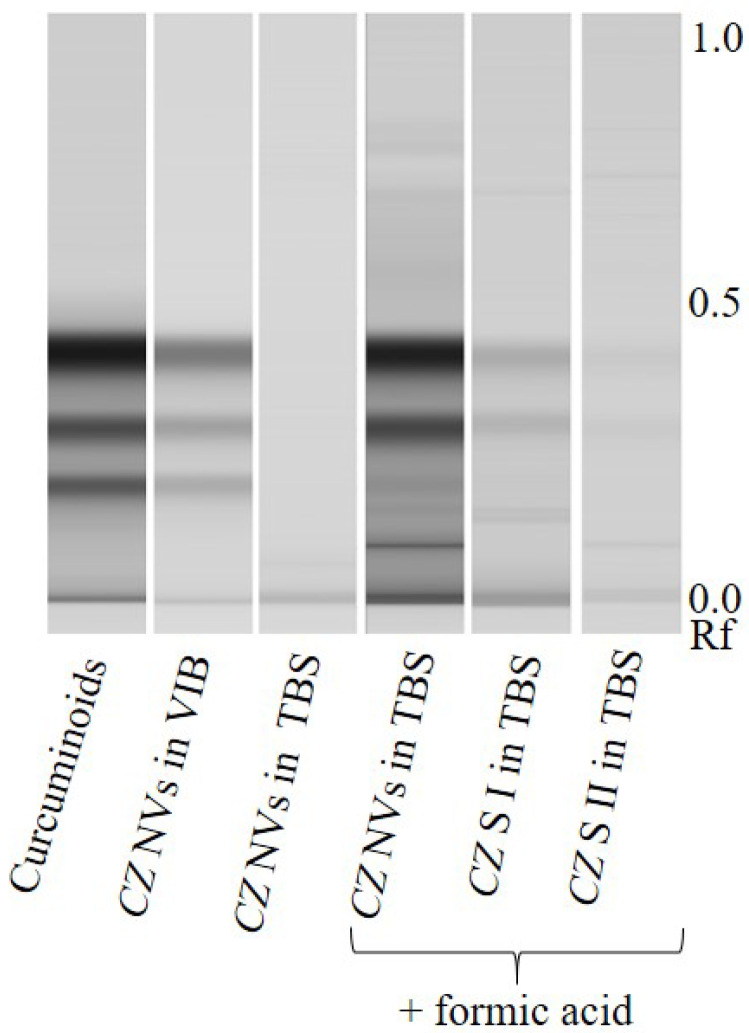
Influence of pH on secondary metabolite lipophilicity by the example of a curcuminoid HPTLC-scan. Nanovesicles (NVs) from *Curcumae zanthorrhizae rhizoma, Curcuma zanthorrhiza* Roxb. (*CZ*) were prepared in acidic vesicle isolation buffer (VIB), as well as in alkaline Tris-buffered saline (TBS). While curcuminoids (curcuminoid mixture contained curcumin, demethoxycurcumin and bisdemethoxycurcumin *, curcumin with the highest Rf, bisdemethoxycurcumin with the lowest) dissolved in the organic extractant, when the vesicles were prepared in acidic VIB, vesicles in TBS had to be acidified, since otherwise acidic curcuminoids would not dissolve within the solvent. These results implicate that secondary metabolites likewise cannot pass membranes when the buffer is chosen to generate charges in the structures of interest. Rf: Retention factor S: Supernatant of high speed centrifugation before (I) and after (II) washing the nanovesicles. * The absorbance spectrum of the band corresponding to bisdemethoxycurcumin in lane “*CZ* NVs in VIB” showed severe differences to curcuminoid spectra and was possibly caused by caffeic acid derivatives.

The TLC-chromatogram of curcuminoids in nanovesicles from *Curcumae zanthorrhizae rhizoma*, *Curcuma zanthorrhiza* Roxb. (Figure 3) shows the influence of pH on curcuminoid solubility in ethyl acetate. While curcuminoids dissolved in the organic solvent when nanovesicles were prepared in acidic VIB, the deprotonated form present in alkaline TBS had to be acidified prior to extraction. Figure 3 further indicates that curcuminoids were enriched in Javanese turmeric nanovesicles compared to the aqueous supernatants and that the washing step efficiently removed soluble contaminants.

**Figure 4:**
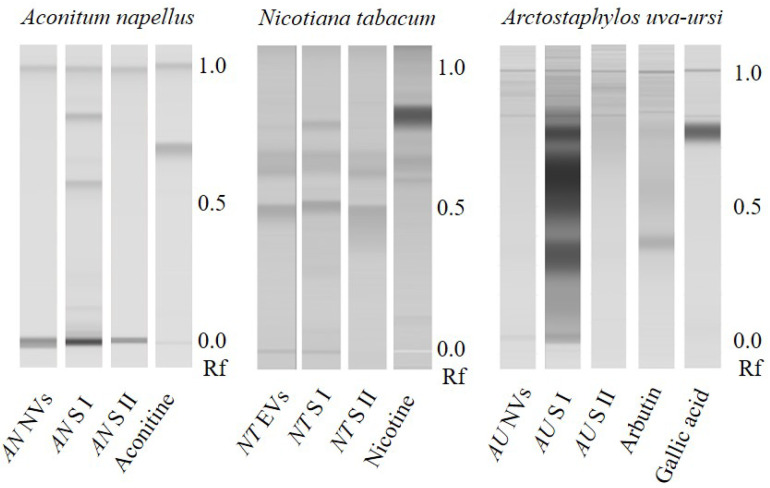
HPTLC-scans of nanovesicles (NVs) isolated from dried *Aconiti tuber*, *Aconitum napellus* L. (*AN*), extracellular vesicles (EVs) from *Nicotiana tabacum* L. (*NT*) leaves’ apoplastic fluid and NVs from and *Uvae-ursi folium*, *Arctostaphylos uva-ursi* (L.) Spreng. (*AU*). While the aqueous supernatants of high-speed centrifugation contained characteristic secondary metabolites prior to the washing step (S I) in all three samples, those structures have not been found in the investigated vesicle samples. Nonetheless, the metabolites present in S I have been effectively removed from the vesicles during the washing step, as the chromatograms of the second supernatants (S II) show. Interestingly, *AN* S I showed no aconitine band but instead two bands giving a positive reaction with Dragendorff’s reagent and similar absorbance spectra like aconitine. LC-MS analysis of reextracted silica identified aconitine to be present in traces, besides larger amounts of hypaconitine and mesaconitine. Rf: Retention factor.

After this first indication that secondary metabolites could be found in plant nanovesicles, we also investigated nanovesicles from dried *Aconiti tuber, Aconitum napellus* L. and *Uvae-ursi folium*, *Arctostaphylos uva-ursi* (L.) Spreng. as well as EVs from *Nicotiana tabacum* L. leaves’ apoplastic fluid for the occurrence of characteristic compounds (alkaloids and phenolics). Chromatograms in Figure 4 show that neither of the investigated vesicle sample contained detectable amounts of the respective analyte, while characteristic metabolites were determined in each aqueous supernatant of the first high speed centrifugation step (S I).

**Figure A1:**
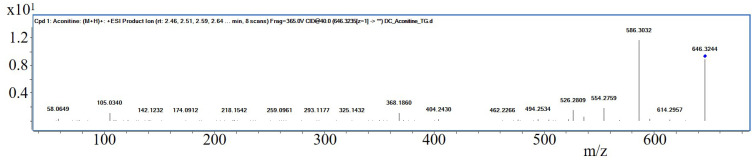
Product ion spectrum of aconitine standard.

**Figure A2:**
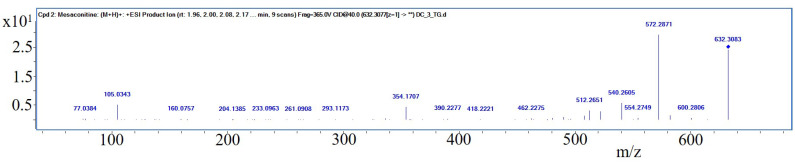
Product ion spectrum of putative mesaconitine.

**Figure A3:**
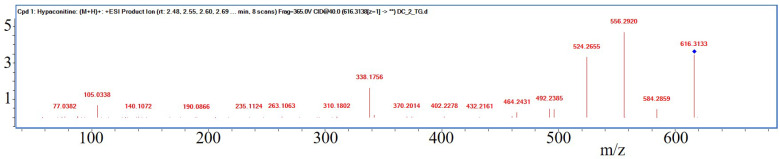
Product ion spectrum of putative hypaconitine.

Interestingly, S I of *Aconiti tuber* contained only traces of aconitine, while the two prominent bands in the relating track have been identified putatively being hypaconitine and mesaconitine by liquid chromatography—mass spectrometry (LC-MS, PharmaMS, Core Facility BioSupraMol, Freie Universität Berlin, Germany) after reextracting the excised bands from the HPTLC plate. Aconitine was used as positive control. Fragments (For product ion spectra see Appendix A, Figure A1, Figure A2 and Figure A3) appeared to confirm the identity of the norditerpene alkaloids (The absorbance spectra correlation between aconitine reference and the two derivatives was 97.6% and 98.9% and the substances showed positive reactions with Dragendorff’s reagent.) according to [33]:

- Aconitine *m/z* 646 → 586
- Mesaconitine *m/z* 632 → 572
- Hypaconitine *m/z* 616 → 556

**Figure 5:**
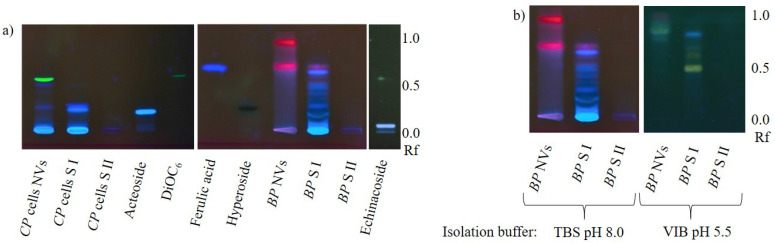
(**a**) HPTLC-chromatogram at 366 nm of *Craterostigma plantagineum* Hochst. (*CP*) nanovesicles (NVs) from in vitro cultured cells and from dried *Betulae folium*, *Betula pubescens* Ehrh. and/or *Betula pendula* Roth (*BP*) and related supernatants (S) of NV isolation before (I) and after (II) washing vesicles. Similar to the membrane dye DiOC_6_, lipophilic chlorophylls appeared to be enriched in the vesicle fraction. While no blue-fluorescing zones occurred in birch NVs, those from *C. plantagineum* showed such zones, probably due to caffeic acid derivatives that could make their way into the vesicles because of the acidic pH of the cell suspension growth medium. (**b**) Influence of buffer pH on secondary metabolites in NVs at 366 nm. While phenylpropanoids were not found in NVs if isolated under alkaline conditions, preparation in an acidic environment resulted in the detection of lipophilic yellow- and blue-fluorescing substances, probably aglyca of flavonoids and caffeic acid derivatives. DiOC_6_: 3,3’-dihexyloxacarbocyanine iodide; Rf: Retention factor; TBS: Tris-buffered saline; VIB: vesicle isolation buffer.

Phenylpropanoid analysis in nanovesicles from *Craterostigma plantagineum* Hochst. cells and NVs from dried *Betulae folium*, *Betula pubescens* Ehrh. and/or *Betula pendula* Roth showed results that appeared to be ambiguous at first sight (Figure 5a). While the blue-fluorescing zones in *C. plantagineum* nanovesicles indicated the occurrence of caffeic acid derivatives in this sample, similar bands were not detected in birch nanovesicles. Instead, nanovesicles from birch leaves contained red-fluorescing lipophilic substances, probably chlorophylls. Considering the fact that chlorophylls are physiologically mainly present in chloroplasts jointly with the above-reported results, we hypothesize that secondary metabolites (or comparable molecules of the primary metabolism) are not actively packaged into plant EVs. Further, lipophilic structures appear to be enriched in vesicle membranes, similarly to the membrane dye 3,3’-dihexyloxacarbocyanine iodide (DiOC_6_). Chlorophylls were likely released due to chloroplast rupture during herb drying.

However, caffeic acid derivatives should not occur in *C. plantagineum* nanovesicles if our theory was consistently valid, but taking the pH of the growth medium of the plant cell culture into account, the hypothesis still stands. The medium pH of 5.8 would allow caffeic acid derivatives to pass vesicle membranes. Regarding the pk_a_ of caffeic acid (pk_a_ = 4.62, referring to https://pubchem.ncbi.nlm.nih.gov/compound/Caffeic-acid#section=pKa (accessed on 2 March 2021)), around 7% of the molecules would be present in the protonated neutral form, which is able to make its way into the vesicle by diffusion and establish equilibrium. This assumption is underlined by the findings presented in Figure 5b. The chromatograms of birch nanovesicles and related supernatants from the isolation process, in context with the pH of buffer used for the vesicle isolation have been compared. As expected, isolating the vesicles in a slightly acidic environment resulted in the presence of phenylpropanoids in the nanovesicle sample. The high retention factor (Rf) of the yellow- and blue-fluorescing zones, which were likely caused by flavonoids and caffeic acid derivatives, demonstrate their hydrophobicity letting us assume, that mainly aglyca were enriched here, not glycosides.

### 2.3. Proteomics

Protein identification from *N. tabacum* leaf apoplastic fluid-derived EVs was troubled by ribulose-1,5-bisphosphate carboxylase-oxygenase (RuBisCO). This extremely highly abundant enzyme—probably the most abundant protein in nature [34]—appeared as a contamination throughout the whole lane of the sodium dodecyl sulfate—polyacrylamide gel electrophoresis (SDS-PAGE) of isolated EV samples, severely impairing the identification of additional proteins in excised bands via peptide mass fingerprint and matrix assisted laser desorption ionization—time of flight—mass spectrometry (MALDI-TOF-MS). Nonetheless, two proteins could be identified: 40S ribosomal protein S4 ≈ 30 kDa and 40S ribosomal protein S6 ≈ 22 kDa (both confirmed by MS-MS data). Related proteins have already been reported in plant nanovesicles [10,13,14,19] but they do not appear to be very specific for plant EVs.

As a consequence of the RuBisCO interference, another approach towards protein identification was made with EVs from *N. tabacum* in vitro cell culture medium, this time isolated by TFF. Here, the ≈85 kDa hydrolase β-xylosidase/α-L-arabinofuranosidase 2-like protein (confirmed by MS-MS, apparently a mixture of two or more isoforms) was reliably identified as an EV protein.

Following the thread of proteomics, *C. plantagineum* EVs from in vitro cell culture medium and nanovesicles from homogenized cells were also investigated using a LC-MS-based approach. We identified proteins in two batches of EVs and nanovesicles isolated from *C. plantagineum* cell suspension cultures. The two batches differ in the amount of input material for proteomics—the second batch allowed the identification of more proteins exclusively present in EVs or nanovesicles. The results of the first and second batch were compiled in Supplementary Materials, respectively and selected results in Table 1. The complete MS proteomics datasets are available via ProteomeXchange with the identifier PXD024203 via http://www.ebi.ac.uk/pride/archive/projects/PXD024203 (accessed on 2 March 2021).

### 2.4. Phospholipid Profiles

In order to validate or refute our hypothesis that the phospholipid composition influences the uptake of nanovesicles into certain cell types or specific organs, two main aspects have to be taken into account: (i) To which extent do the lipid compositions of plant nanovesicles vary? (ii) Are these variations causally linked to any cellular uptake preferences?

To investigate the first aspect, we used an HPTLC method to get a first insight into plant phospholipid profiles. Preliminary investigations showed that sterols (β-sitosterol und stigmasterol) were not retained by the stationary phase and retention factors resulted to be nearly 1. Further, the HPTLC of 1,2-dioleoyl-*sn*-glycero-3-phosphocholine and 2-oleoyl-1-palmitoyl-*sn*-glycero-3-phosphocholine has shown no differences in retention factors (data not shown). Thus, the phospholipid class apparently influences the migration distance of the lipid more than individual fatty acid residues.

**Figure 6:**
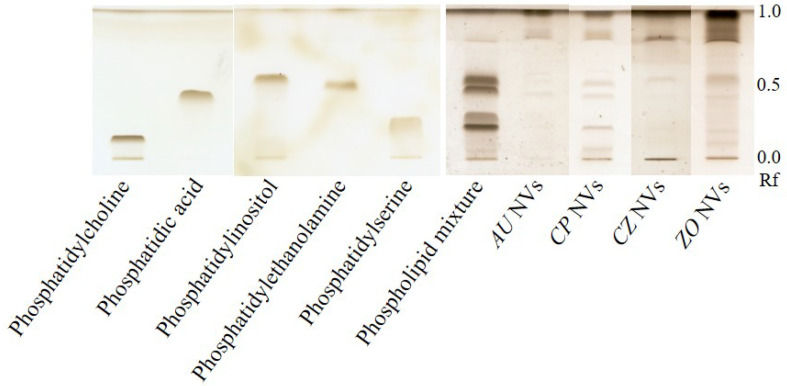
HPTLC phospholipid profiles of methanol-chloroform extracts from nanovesicles isolated from *Uvae-ursi folium, Arctostaphylos uva-ursi* (L.) Spreng. (*AU*), *Craterostigma plantagineum* Hochst. (*CP*) cells, *Curcumae zanthorrhizae rhizoma, Curcuma zanthorrhiza* Roxb. (*CZ*) and *Zingiberis rhizoma, Zingiber officinale* Roscoe (*ZO*) after derivatization with 10% (*m*/*v*) CuSO_4_ in 8% (*v*/*v*) H_3_PO_4_ and 10–15 min heating to 140–145 °C, charring analytes. Rf: Retention factor.

Although the application of single phospholipids provided different retention factors (Figure 6), phosphatidylethanolamine and phosphatidylinositol were not satisfactorily separated when the five phospholipid classes were applied together. Nonetheless, the method was used to get a first insight into the phospholipid profiles of a row of nanovesicle extracts and as the chromatogram in Figure 6 shows, we were able to identify phospholipids in these samples as follows:

- *Uvae-ursi folium* nanovesicles: Phosphatidic acid
- *C. plantagineum* nanovesicles: Phosphatidylcholine, Phosphatidic acid, Phosphatidylethanolamine
- *Curcumae zanthorrhizae rhizoma* nanovesicles: Phosphatidylinositol
- *Zingiberis rhizoma* nanovesicles: Phosphatidic acid, Phosphatidylethanolamine, Phosphatidylinositol

Our investigations also revealed that relatively large amounts of vesicles were necessary to achieve analyzable chromatograms. 1–2 μg of each phospholipid were clearly detectable. Meanwhile, a minimum of 1 mg (calculated as total protein amount) of a vesicle sample had to be extracted. Therefore, other investigated isolates (e.g., nanovesicles from *Aconiti tuber*, *Betulae folium* and *Uvae-ursi folium*) remained without reportable results.

## 3. Discussion

Beyond doubt, the research on EVs from all domains of life has already revolutionized our understanding of intercellular information transmission, but, in particular with regard to plant-derived EVs, its potential is still waiting to be fully tapped. With our present investigation we intended to keep plant nanovesicles in the scientific focus and move a little closer towards the usage of these promising structures. Our finding that plant EVs can be isolated from plant in vitro culture media might pave the way towards that aim.

Compared to the vesicle isolation process from conventionally grown and harvested plant materials, the amount of accompanying substances would be minimized when cell culture media were used as starting point. Besides, the production could take place under controlled sterile environmental conditions, which ought to ensure product consistency and maybe plant cells could even be induced, by additives, to produce EVs with desired properties, or loaded with drugs by plant cells themselves. Differences between the metabolisms of humans and plants could even allow the application of drugs that cannot be packaged into vesicles in in vitro cultures of human cells (e.g., due to cytotoxic effects). However, before this perspective can be translated into first studies, more knowledge on plant nanovesicles must be compiled. We therefore gathered information on secondary metabolites, the proteome and lipids of plant EVs and nanovesicles in the investigation at hand.

### 3.1. Secondary Metabolites

Due to the complexity of nanovesicle samples containing nucleic acids, proteins, lipids and salts from isolation buffers, HPTLC appeared to be the chromatography technique of choice, since it is especially robust, yet sensitive and can be applied for a broad set of analytes. Secondary metabolite analysis in nanovesicles from several plant species revealed that especially lipophilic molecules were vesicle-associated. As such, we found curcuminoids and chlorophylls being enriched in the investigated vesicles, just like DiOC_6_, if the membrane dye had been added. Meanwhile, neither alkaloids nor phenols or phenylpropanoids appeared to be typically associated with the isolated vesicles. Analogously, Stremersch et al., 2016 reported that cholesterol-modified siRNA bound to the vesicle membrane surface with the cholesterol residue acting as anchor [35]. We therefore conclude that secondary metabolites are associated to nanovesicle membranes in a passive manner due to lipophilicity, rather than being actively packaged into the vehicles.

The above-mentioned ambiguous data about secondary metabolites in plant nanovesicles are not in contradiction with our theory, since shogaol, which has been found in ginger nanovesicles [22], is chemically and biogenetically closely related to curcuminoids. The reports about flavonoids are not consistent since naringin and naringenin were found in grapefruit nanovesicles [30] but ascorbic acid as well as naringenin were absent from nanovesicles from orange [24]. Howsoever, in the acidic milieu of citrus fruits, we do not question the possibility of diffusion-driven flavonoid distribution through all compartments.

From an evolutionary point of view, thinking that secondary metabolites are not actively packaged into EVs is not odd. The high conservation of EV formation and the fact that practically every living cell secretes vesicles [36], makes it appear likely that mechanisms of EV formation had been established in eukaryotic metabolism prior to the development of plant secondary metabolite production.

What still remains puzzling is the extent to which plant cells may vary EV compositions. Similar to what is known from the animal kingdom [37], plants could tune EVs as a response to certain physiological or environmental conditions. As such, approaching stimuli might induce the formation of vesicles aligned to meet the requirements of the particular signal.

Among the objectives of further investigations could be a more comprehensive screening of a greater variety of species and analytes, together with a clustering of *n*-octanol-water partition coefficients of vesicle-associated substances and the evaluation of differences between aglyca and glycosides. A possible attempt to prove our hypothesis might also be to grow plant cell suspension cultures in media with different pH ranges and analyze EVs for secondary metabolites.

### 3.2. Proteomics

The high abundance of RuBisCO hindered the identification of lower-abundance proteins, underlining the importance of a careful evaluation of the results of proteomic investigations. Additionally, reconfirmation is required to clarify whether the proteins are indeed structural EV components and not just co-isolated. Considering this constraint, the list of proteins in Table 1 was thoughtfully selected from the proteins identified in EVs or NVs but not in the supernatants S I and S II of high-speed centrifugation.

It is remarkable that the hydrolase β-xylosidase/α-L-arabinofuranosidase 2-like was found in the vesicles of both investigated species *C. plantagineum* and *N. tabacum*. This enzyme is involved in cell wall remodeling [38,39] and might be responsible for the passage of EVs through cell walls, which has not yet been conclusively elucidated [13]. One aspect of the hypothetical passage mechanisms is based on the assumption that EVs are associated with enzymes loosening the cell wall structure. The identified hydrolase could be one of these enzymes. Guerra-Guimarães et al., 2014 found this hydrolase to be prominently abundant in apoplastic fluid from *Coffea arabica* L. leaves [38]. Regarding that in this work proteins were concentrated by ultrafiltration, apoplastic EVs might have been concentrated unintentionally.

Besides β-xylosidase/α-L-arabinofuranosidase 2-like, we identified a couple of other proteins involved in cell wall reorganization, many of them exclusively in the vesicle fraction (Table 1). Interestingly, even an endochitinase EP3-like was detected, indicating the relevance of EVs as defensive agents against pathogenic fungi.

In order to estimate cellulase-/hemicellulase activity, zymograms could perspectively help to evaluate enzyme activity and thus the extent to which hydrolases are correlated to the vesicles themselves or rather surrounded by them. If the vesicle fraction appears to be active, we might have a marker protein at hand. Other potential marker proteins are listed in Table 1 “Reported in plant NVs or EVs”.

Another interesting finding is the joint appearance of transmembrane 9 superfamily member 11, AP-complex subunits and membrane steroid-binding protein 2, as well as proteins that are related to ubiquitination. The presence of these proteins indicates that the isolated vesicles might originate not only from endosomal pathways with Golgi-apparatus involvement, but also directly from the plasma membrane, hinting at the correctness of our hypothesis that plant cells secrete exosomes, as well as microvesicles, in a similar manner as animal cells do.

### 3.3. Phospholipid Profiles

Plant biomembranes consist of glycerolipids, sphingolipids and sterols [40]. Among glycerolipids, the phospholipid group appears to play a crucial role not only for nanovesicle stability but probably also for addressing target cells. Although the accuracy of our method can yet be improved, this technique is broadly applicable and high throughputs are possible. The observed need of substantial sample amounts has been reported similarly by other groups [18,41]. Despite these obstacles, we were able to get a first insight into the phospholipid profiles of nanovesicles from a panel of different plant species.

Creating such lipid profiles of a variety of nanovesicles from the plant kingdom might improve our understanding of EV and nanovesicle formation, addressing and uptake, as well as related synthetic nanocarriers. If we were once able to address specific target cells with biocompatible drug-loaded nanocarriers, side effects could be brought down to an absolute minimum. Comparative studies of nanovesicles and EVs from one and the same plant, like recently published by Liu et al., 2020 [1], may also help improve our knowledge, probably even beyond the fields of lipids and cellular uptake.

## 4. Materials and Methods

### 4.1. Isolation of Nanovesicles and EVs

Plant EVs can be obtained from apoplastic fluid [12,13] but in the investigation at hand, we also isolated EVs from plant cell culture media. Moreover, nanovesicles were isolated from homogenized plant materials, such as dried powdered herbs or from in vitro cultured plant cells. For vesicle rehydration, 50 g dried herbs were incubated overnight in 500 mL of the respective buffer (see Section 4.3) at 4 °C. In vitro cultured plant cells were ground with ice-cooled mortar and pestle and PBS added respectively. The suspensions of either cultured cells or herb powder were decanted and centrifuged differentially. All centrifugation steps were performed at 4 °C. The aqueous extracts were centrifuged twice at 4000× *g* for 10 min to remove large particles (Beckman Allegra X 30 R centrifuge, SX 4400 rotor; Beckman Coulter, Brea, CA, USA). The supernatant was then centrifuged 15 min at 20,000× *g* removing medium size particles (Avanti J-26 S XP centrifuge, JA 25.50 rotor; Beckman Coulter). The supernatant was filtered through a 0.85 μm syringe filter (Rotilabo^®^ CME, Carl Roth GmbH & Co. KG, Karlsruhe, Germany), membranes optionally stained using DiOC_6_ and then nanovesicles pelleted at 50,000× *g*; 90 min (Avanti J-26 S XP centrifuge, JA 25.50 rotor). The resulting pellet was washed using freshly 0.2 μm filtered buffer and the final centrifugation step repeated. The pellet, containing isolated nanovesicles, was suspended in 100–1000 μL filtered buffer, depending on the yield. After isolation, protein concentrations of the vesicle samples were determined by Roti^®^Nanoquant Bradford assay (Carl Roth) according to the manufacturer’s manual.

For the article at hand, plant EVs were isolated from the apoplastic fluid of *Nicotiana tabacum* L. (tobacco seeds were provided by the Botanical Garden Berlin, Germany; accession number 107-01-95-14, and grown in the greenhouse at the Institute of Pharmacy, Freie Universität Berlin, Germany). Apoplastic fluid was obtained as previously described [42] according to Rutter and Innes 2016 [13] using the vacuum-infiltration centrifugation technique.

EVs were further concentrated from suspension culture media of *Craterostigma plantagineum* Hochst. and *N. tabacum*. The *C. plantagineum* calli were dedifferentiated from leaves of plants supplied by Prof. Dr. Dorothea Bartels (Rheinische Friedrich-Wilhelms-Universität Bonn, Germany) and cultured as described previously [43]. Cell cultures were grown for 2 weeks under light in 2 L flasks at 100 rpm and 26 °C.

Nanovesicles were isolated from the above-mentioned cultured plant cells, as well as from dried herbs of: *Aconitum napellus* L., *Arctostaphylos uva-ursi* (L.) Spreng., *Betula pubescens* Ehrh. and/or *Betula pendula* Roth, *Curcuma zanthorrhiza* Roxb. and *Zingiber officinale* Roscoe (all purchased from Alfred Galke GmbH, Bad Grund, Germany).

Suspension culture media and cells of *N. tabacum* were kindly provided by the Department of Pharmacognosy and Herbal Medicines of Wrocław Medical University, Wrocław, Poland.

### 4.2. Electron Microscopy

Isolated nanovesicles were visualized by TEM, using the scanning electron microscope Hitachi SU 8030 in TEM mode (Hitachi Ltd., Tokyo, Japan). Samples were prepared as earlier described [42]: 5 μL of each sample were placed on 300 mesh Formvar and carbon-coated copper grids and incubated 1–2 min. Grids were then rinsed and negatively stained by pipetting ≈80 μL Uranyless^®^ (Science Services GmbH, Munich, Germany) across the grid surface. Fluid excess was carefully blotted using Kimwipe before overnight drying in a desiccator. TEM imaging was performed using 30 kV acceleration voltage.

### 4.3. Secondary Metabolites

The buffer for vesicle isolations has been selected regarding the secondary metabolites that ought to be investigated. For the analytics of alkaline structures, VIB was used—a MES buffer of pH 5.5 (20 mM MES, 100 mM NaCl, 10 mM EDTA, 10 M NaOH to adjust pH to 5.5), while we chose TBS of pH 8.0 (100 mM Tris, 100 mM NaCl, 10 mM EDTA, 10% HCl to adjust pH to 8.0) when acidic metabolites were in the focus of investigation. This way, we intended to inhibit the membrane passage of secondary metabolites—alkaloids would be positively charged due to protonation in acidic environment and acidic compounds were negatively charged because of deprotonation under basic conditions.

To evaluate the content of secondary metabolites, 200 μL of each sample have been lyophilized (Alpha 1–2 LDplus, Martin Christ GmbH, Osterode am Harz, Germany) and afterwards extracted in 90 μL of an organic solvent (e.g., ethyl acetate or a mixture of equal parts methanol and chloroform). Due to the pH of buffers used for vesicle isolation, secondary metabolites had to be neutralized prior to extraction. Therefore, 10 μL of formic acid were added to protonate acidic molecules while 10 μL of 10 M NaOH were added deprotonating alkaloids. After thorough mixing and brief centrifugation in a benchtop centrifuge (Heraeus Biofuge pico, Heraeus GmbH, Hanau, Germany) to settle undissolved solids at the bottom of the reaction tube, 5–10 μL of supernatants were applied band-shaped onto HPTLC plates (Nano-SIL-20/UV_254_, Macherey-Nagel GmbH & Co. KG, Düren, Germany) by Linomat IV (Camag AG, Muttenz, Switzerland). Alkaloid HPTLC development was performed automated in an AMD2 device (Camag) following the gradient in Appendix A, Table A1. Plates for acidic metabolite analysis were also developed automated, using ADC2 (Camag). Mobile phase compositions are listed in Appendix A, Table A2.

After development, plates were documented under UV light at 254 and 366 nm and scanned by TLC Scanner 4 (Camag) at 210, 250 and 280 nm at first. These first scans were used to create absorption spectra of selected bands and optimize the detection wavelength of further scans to the maxima of the analytes of interest. Identities of bands with similar Rf were confirmed or disproved by matching the absorption spectra. After the described densitometric analysis, plates were eventually derivatized with Dragendorff’s reagent or 5% 2-aminoethyl diphenyl borate in methanol.

### 4.4. Proteomics

Proteins of plant extracellular- and nanovesicles have been identified from leaves’ apoplastic fluid or suspension cultured cells of the two plants *N. tabacum* and *C. plantagineum*. The vesicle samples were usually isolated by differential centrifugation as described above, while tobacco EVs from cell culture medium were isolated using a tangential flow filter (TFF-Easy, HansaBioMed Life Sciences Ltd., Tallinn, Estonia) according to the manufacturer’s instructions. For proteomic investigations, 0.5 mM phenylmethylsulfonyl fluoride (PMSF) were freshly added to the isolation buffer.

To separate proteins by SDS-PAGE, one volume of isolated tobacco EVs was mixed with 4 volumes of reducing 4x Laemmli-buffer (Bio-Rad Laboratories Inc., Hercules, CA, USA) and proteins denatured by boiling at 95 °C for 10 min. The separation itself was performed by discontinuous SDS-PAGE, conducted following the instructions of Jansohn and Rothaemel 2012 [44] using 12.5% (*w*/*v*) polyacrylamide resolving gel and 5% (*w*/*v*) polyacrylamide stacking gel on top. 15–20 μL of each sample were added to gel pockets and electrophoresis performed at 200 V for approximately 45 min, until bromophenol blue reached the bottom of the gel. After electrophoresis, gels were immediately transferred into the staining solution. According to Neuhoff et al., 1988 [45], gels were stained overnight in 0.1% (*w*/*v*) Coomassie Brilliant Blue G-250, 2% (*v*/*v*) H_3_PO_4_, 10% (*w*/*v*) (NH_4_)_2_SO_4_, and 20% (*v*/*v*) methanol. The next day, gel matrices were destained using 25% (*v*/*v*) methanol.

Out of these gels, visible protein bands were excised and proteins in-gel digested with trypsin (for MS from porcine pancreas, SERVA Electrophoresis GmbH, Heidelberg, Germany) according to Shevchenko et al., 1996 [46]. Prior to trypsin digestion, proteins were destained, reduced and carbamidomethylated. Digestion supernatants were then investigated by MALDI-TOF-MS (Ultraflex-II TOF/TOF, Bruker Daltonics, Bremen, Germany) with *α*-cyano-4-hydroxycinnamic acid as matrix. Database searches for protein identification of the peptide mass fingerprints were performed using Mascot (Matrix Science Ltd., London, UK, http://www.matrixscience.com (accessed on 2 March 2021)) against the SwissProt database (all entries). The mass tolerance was set at ±75 ppm and we allowed for one missed cleavage. Carbamidomethylation (CAM) of cysteines was set as a fixed modification, and oxidation of methionines as variable modification. The identifications were confirmed by MS/MS sequencing of selected peptides acquired in the LIFT mode [47]. Here fragment peptide mass tolerance was set at ±100 ppm and fragment mass tolerance at ±0.7 Da.

Following up on these preliminary proteomic investigations, the methodology has been changed to a more comprehensive protocol using nano LC-MS/MS, investigating *C. plantagineum* samples from cell culture as follows:

Lyophilized *C. plantagineum* samples were directly denatured in fresh lysis buffer (urea 7 M, thiourea 2 M, 3-[(3-cholamidopropyl)dimethylammonio]-1-propanesulfonate 0.5% (*w*/*v*)) and subsequently loaded on a precast gel (Criterion™ XT precast 1D gel 12% Bis-Tris, Bio-Rad) for a short migration. Proteins were stained with Instant Blue (Gentaur BVBA, Kampenhout, Belgium), reduced and alkylated. Thereafter, proteins were digested with trypsin 5 ng/µL (sequencing mass grade, Promega) o/n at 37 °C. The extracted peptides were solubilized to perform protein identification by nano LC-MS/MS, using a NanoLC-425 Eksigent system coupled to TripleTOF^®^ 6600+ MS (SCIEX, Darmstadt, Germany). The protocol is essentially the one described previously [48].

The MS data were processed with Mascot (version 2.4.2) using Mascot Daemon interface (version 2.4.2, Matrix Science) by searching against an in-house annotated database of *C. plantagineum* (288,270 sequences). The parameters were set as follows: peptide tolerance of 20 ppm, fragment mass tolerance of 0.5 Da, maximum two missed cleavages, CAM of cysteine as fixed modification and oxidation of methionine, N-terminal protein acetylation, N-terminal glutamine to pyroglutamate and tryptophan to kynurenine as variable modifications. Only the proteins identified with a significance Mascot-calculated threshold corresponding to a *p*-value < 0.05 and at least two sequences per protein and one unique sequence per protein were accepted.

### 4.5. Phospholipid Profiles

According to the methods of Mu et al., 2014 [18] and Deranieh et al., 2013 [49], we established an instrumental HPTLC method to generate profiles of vesicle phospholipids: after vesicle isolation, distinct protein amounts of vesicle samples were lyophilized in glass tubes, avoiding any possible adsorption of lipids to plastic tubes. After adding adequate volumes of a mixture of equal parts methanol and chloroform (≈100–150 μL), samples were thoroughly mixed and then centrifuged for 5 min at 4000× *g* (Beckman Allegra X 30 R centrifuge, SX 4400 rotor). 5 and 10 μL of the supernatant solvent (Combining secondary metabolite analysis and lipid profiling was also possible.) were applied band-shaped to HPTLC plates (Nano-SIL-20/UV_254_) using Linomat IV and developed in the AMD2 device. The mobile phase gradient is given in Appendix A, Table A3. Detection of phospholipids was not possible by densitometric analysis, though HPTLC plates were bathed in 10% (*m*/*v*) CuSO_4_ in 8% (*v*/*v*) H_3_PO_4_ and afterwards heated to 140–145 °C, charring analytes [50].

## 5. Conclusions

In the present study, a simple and easily scalable protocol is described to isolate plant EVs and nanovesicles together with data on proteins, secondary metabolites and lipid compositions of EVs and nanovesicles from different plant species. The protocol proved its validity in extracting EVs and nanovesicles from a variety of starting materials, such as plant tissues, undifferentiated cells and in vitro cell culture medium. The successful isolation of EVs from plant cell culture medium offers tremendous potential for plant bioprocess engineering whereby growth conditions in bioreactors may be optimized for specific plant cell lines and elicitation protocols fine-tuned. Indeed, we believe that elicitation will be an effective tool to boost the production of EVs in plant cell cultures, as they are known to stimulate a defense response.

## Figures and Tables

**Table 1:** Proteins determined in extracellular vesicles (EVs) from medium and nanovesicles (NVs) of in vitro cultured cells of *Craterostigma plantagineum* Hochst. (excerpt from Supplementary Materials). The listed proteins have been selected from the set of proteins that were identified with at least one unique sequence per protein in EVs or NVs but absent from the supernatants S I and S II of high-speed centrifugation.

GI Accession	Mascot Protein Score	Number of Significant Unique Sequences	Description	Comments	Ref.
2440044	41	2	plasma membrane intrinsic protein 1C	Aquaporin	
747090550	195	2	aquaporin PIP2-7-like		
1102288605	5211	8	beta-galactosidase 10	Cell wall related	
747099132	3135	6	glucan endo-1,3-beta-glucosidase 6		
747098362	3020	5	probable pectinesterase/pectinesterase inhibitor 51		
1111022911	2389	8	glucan endo-1,3-beta-glucosidase 6		[13]
747064393	2356	6	glucan endo-1,3-beta-glucosidase 8-like		
848910120	1858	4	probable pectinesterase/pectinesterase inhibitor 51		
1173765936	1819	4	probable polygalacturonase		
747044150	1202	2	glucan endo-1,3-beta-glucosidase 4		
747058516	989	3	PLASMODESMATA CALLOSE-BINDING PROTEIN 3-like		
747049508	264	2	fiber protein Fb34		
747098951	90	2	probable glucan 1,3-beta-glucosidase A		
747052794	67	3	beta-galactosidase 17		
976918650	64	2	vesicle-associated membrane protein 722		
697141632	54	2	Beta-galactosidase 17		
1219121472	52	1	polygalacturonase inhibitor protein		
698520102	45	2	glucan endo-1,3-beta-D-glucosidase		
848878290	34	2	alpha-L-arabinofuranosidase 1		
1109239080	40	2	heat shock 70 kDa protein 15-like	Chaperone	
1102140324	68	2	protein unc-45 homolog A		
747097466	17,016	4	berberine bridge enzyme-like 21	Oxidative reaction *	
848927236	5502	7	reticuline oxidase-like protein		
747097466	252	3	reticuline oxidase-like protein		
747048987	418	7	annexin D5-like	Reported in plant NVs or EVs	
747075688	413	4	clathrin heavy chain 1-like		
848877390	98	2	coatomer subunit alpha-1-like		
747061404	96	2	coatomer subunit beta-1		[14,19]
848918315	168	4	coatomer subunit beta’-2-like		
747082147	212	4	coatomer subunit gamma		
1024019175	122	2	patellin-3-like isoform X2		[14]
747083940	891	2	tetraspanin-3-like		
747047721	2398	2	tetraspanin-8-like		[8,13]
1102146030	11,629	8	endochitinase EP3-like	Stress response	
222862882	48	2	G-type lectin S-receptor-like serine/threonine-protein kinase At1g34300		

* alkaloid biosynthesis; EVs: extracellular vesicles NVs: nanovesicles.

## Data Availability

The data presented in this study are available in the Supplementary Tables S1 and S2 and have been deposited to the ProteomeXchange Consortium via the PRIDE [51] partner repository with the dataset identifier PXD024203 at http://www.ebi.ac.uk/pride/archive/projects/PXD024203 (accessed on 2 March 2021).

## Supplementary Materials

The Supplementary Materials are available at https://www.mdpi.com/article/10.3390/ijms22073719/s1.

## Abbreviations

The following abbreviations are used in this manuscript:

CAM	carbamidomethylation
DiOC_6_	3,3’-dihexyloxacarbocyanine iodide
EV	extracellular vesicle
LC-MS	liquid chromatography-mass spectrometry
MALDI-TOF-MS	matrix assisted laser desorption ionization–time of flight–mass spectrometry
MES	2-(*N*-morpholino)ethanesulfonic acid
MVB	multivesicular body
NV	nanovesicle
PMSF	phenylmethylsulfonyl fluoride
Rf	retention factor
RuBisCO	Ribulose-1,5-bisphosphate carboxylase-oxygenase
S I & II	supernatants of high speed centrifugation before (I) and after (II) washing vesicles
SDS-PAGE	sodium dodecyl sulfate-polyacrylamide gel electrophoresis
sRNA	small non-coding RNA
TBS	Tris-buffered saline
TEM	transmission electron microscopy
TFF	tangential flow filtration
VIB	vesicle isolation buffer

## Appendix A

**Table A1:** Composition and solvent fronts of HPTLC gradient for alkaloid analysis.

Ammonia 25%	Ethyl Acetate	Methanol	Water	Solvent Front
5%	-	90%	5%	15 mm
5%	24%	70%	1%	30 mm
5%	55%	40%	-	40 mm
5%	85%	10%	-	50 mm

**Table A2:** Composition of mobile phases for acidic secondary metabolite analysis.

Curcuminoids	Phenols	Phenylpropanoids
2 vol acetic acid	6 vol formic acid	10 vol formic acid
8 vol toluene	6 vol water	10 vol water
	88 vol ethyl acetate	25 vol 2-butanone
		40 vol ethyl acetate
		15 vol *n*-hexane

**Table A3:** Composition and solvent front of AMD2 HPTLC gradient for phospholipid analysis.

Chloroform	Ethanol	Methanol	Triethylamine	Water	Solvent Front
45%	5%	45%	5%	-	15 mm
40%	12.5%	34%	12.5%	1%	22 mm
30%	20%	28%	20%	2%	29 mm
20%	27.5%	22%	27.5%	3%	36 mm
15%	35%	11%	35%	4%	43 mm
10%	40%	5%	40%	5%	50 mm
